# Recent Progress on Microfluidics Integrated with Fiber-Optic Sensors for On-Site Detection

**DOI:** 10.3390/s24072067

**Published:** 2024-03-24

**Authors:** Weibin Wang, Ling Xia, Xiaohua Xiao, Gongke Li

**Affiliations:** School of Chemistry, Sun Yat-sen University, Guangzhou 510006, China; wangwb33@mail2.sysu.edu.cn (W.W.); xialing@mail.sysu.edu.cn (L.X.)

**Keywords:** microfluidics, fiber-optic sensor, on-site detection, application

## Abstract

This review introduces a micro-integrated device of microfluidics and fiber-optic sensors for on-site detection, which can detect certain or several specific components or their amounts in different samples within a relatively short time. Fiber-optics with micron core diameters can be easily coated and functionalized, thus allowing sensors to be integrated with microfluidics to separate, enrich, and measure samples in a micro-device. Compared to traditional laboratory equipment, this integrated device exhibits natural advantages in size, speed, cost, portability, and operability, making it more suitable for on-site detection. In this review, the various optical detection methods used in this integrated device are introduced, including Raman, ultraviolet–visible, fluorescence, and surface plasmon resonance detections. It also provides a detailed overview of the on-site detection applications of this integrated device for biological analysis, food safety, and environmental monitoring. Lastly, this review addresses the prospects for the future development of microfluidics integrated with fiber-optic sensors.

## 1. Introduction

On-site detection provides qualitative and/or quantitative information on analytes at locations that are usually far from the laboratories. This is crucial in cases where the sample composition may change during transportation, or when results are urgently needed for further action, such as in medical emergencies where accurate therapy dosing is required, in rapidly monitoring environmental contaminants or identifying bacteria in food [1,2]. Traditional experimental operations are typically performed in different functional laboratories, which introduces operational inconveniences and hinders rapid measurement. The achievement of on-site detection mainly depends on the sample pretreatment methods and instrument analysis technologies. The sample pretreatment methods need to be simple and reliable, while the instruments are expected to be handheld, smartphone-based, or at least suitable for use in vehicles [3,4,5,6]. The portability and miniaturization of instruments mean that not all detection methods are suitable for on-site detection.

Microfluidics has been applied in on-site detection, including molecular biology, chemicals analysis, and food safety sensing, due to its advantages in cost, analysis speed, size, and operability [7,8,9]. Microfluidics, as a miniaturized technology, involves the manipulation of fluid samples in microchannels with cross-sectional dimensions in the range of tens to hundreds of microns. It can integrate sample preparation, separation, and detection on a single microchip of a few square centimeters [10,11]. Microchips exhibit unique fluid behaviors due to some special effects, e.g., laminar flow, capillary effect, and rapid heat conduction. Precise fluid control and fast reactions are attributed to these special effects [11]. In recent years, micro-processing technologies, such as micro-milling, laser engraving, and 3D printing, have allowed for the fabrication of microchips with complex structures, enabling flexible combinations and the scalable integration of various operations including separation, concentration, purification, and detection [12,13]. For example, Mishra et al. [14] designed a disposable micro-device that integrated Z-shaped microchannels with micro-lens and optical fiber–coupler structures. This device was effectively used for the label-free detection of heavy metal ions in water, achieving a minimum detectable concentration of 0.5 μg/L. Dong et al. [15] achieved the serial dilution of erythrocytes by repeatedly splitting and mixing the source suspension in bifurcated serpentine microchannels. In addition, our group proposed a microfluidic magnetic spatial confinement strategy, utilizing the straight confined microchannels as enrichment and detection chambers to achieve an ultrasensitive cell immunoassay [16]. The smaller liquid volumes required by these designed structures reduce the reagent consumption and the assay cost. In addition, multiple parallel chemical reactions can be performed, accelerating the outcomes of experiments and research [17].

The commonly used detection methods on microchips are mainly classified into optical detection, electrical detection, and mass spectrometry detection methods [18]. Optical detection has several advantages, such as high analysis speed, non-intrusiveness, and immunity to electromagnetic interference, when compared to other methods. Optical detection can employ the unique optical properties of the target or is reinforced by specific probes, making it ideal for the high-sensitivity on-site detection of targets [19,20,21]. Traditional optical detection on a microchip typically uses a microscope as a central device. Although microchips are small, these bulky and expensive peripheral optical instruments hinder the development of miniaturized devices. This approach has limitations in on-site detection. To address this issue, more attention has been paid to the combination of microfluidics with fiber-optic technology for optical detection due to their irreplaceable advantages, e.g., fast response, low dosage, flexibility, and remote sensing capability [22,23]. Fiber-optic is used for the simultaneous transmission of excitation/collection light. This optical design can overcome the disadvantages of free space optical path and improve the transmission efficiency and detection sensitivity. In addition, optical fibers have the advantage of being easily coated and functionalized, thus allowing a sensor to continuously monitor functionalization and detection, and being small in size and geometrically flexible [24,25].

**Figure 1:**
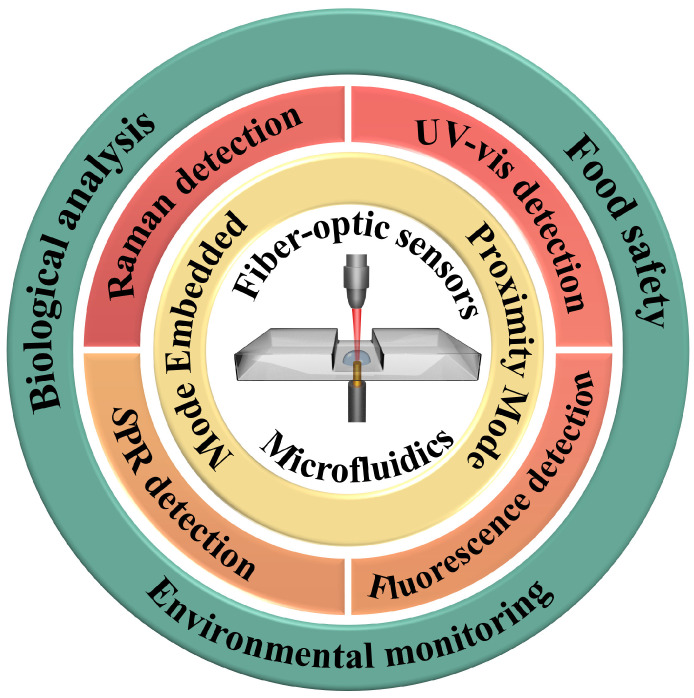
Scheme of microfluidics integrated with fiber-optic sensors used in practical applications.

The integration of microfluidics and fiber-optic sensors enables the separation, enrichment, and detection of samples on a single micro-device, which has the advantages of small size, fast speed, portability, and easy operation. This integrated micro-device combines reliable sample pretreatment with portability in size, making it meet the requirements of the equipment for field detection. This review summarizes the development of microfluidics integrated with fiber-optic sensors over the past decade, mainly focusing on optical detection methods, such as Raman, ultraviolet–visible (UV-vis), fluorescence, surface plasmon resonance (SPR), and localized surface plasmon resonance (LSPR) (Figure 1) [26]. It also highlights the potential applications in on-site detection, e.g., biological analysis, food safety, and environmental monitoring, that have arisen or will arise from this integrated system.

## 2. Optical Detection with Microfluidics Using Fiber-Optic Sensors

### 2.1. Integration Modes

**Figure 2:**
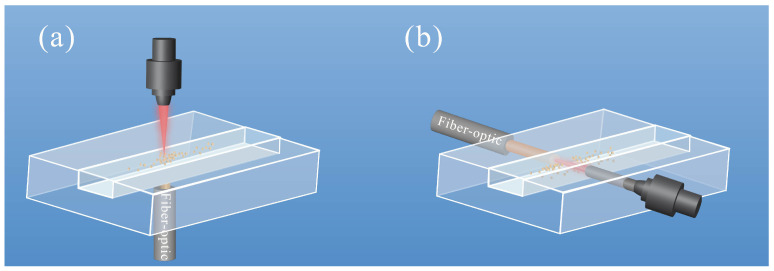
Proximity (**a**) and embedded (**b**) fiber-optic sensors for lab-on-a-chip.

There are two perspectives to classify the fiber-optic sensors on a chip: one is based on the integrated modes on a microchip, and the other is based on its functionality on a microchip. In Figure 2, microchips integrated with fiber-optic sensors are categorized as proximity and embedded modes [27]. The proximity fiber is located close to, but not in direct contact with the microchips (Figure 2a). Though proximity fiber introduces additional noise in the signals, they are easy to assemble on microchips and have been suggested as an economical yet highly sensitive alternative. The embedded fibers allow for optimal performance with minimal signal loss by inserting the fiber into the microchip and ensuring physical contact with the microchannel structure (Figure 2b). However, this integrated mode places higher requirements on the chip processing to ensure a high level of integration between the chip and the fiber. 

In addition, fiber-optic can also be classified into light guides/sensors based on their functionality on microchips: one is assembled on a microchip as a simple light guide to transmit and receive light signals from the target in a flow cell. Two or more fiber-optic sensors can be arranged in a series or parallel sequence on microchips without any modification to the fiber-optic sensors themselves. The other type is modified to create a new sensing element that is located within the microchannel itself [28,29]. The sensing detection of fiber-optic sensors is achieved by adding a series of functional materials, e.g., nanomaterials and metal films, to generate optical effects on the fiber-optic surface, e.g., surface-enhanced Raman scattering (SERS) and SPR/LSPR effects, which have expanded the applications of fiber-optics in analytical chemistry.

### 2.2. Optical Detection

There is growing interest in utilizing the characteristics and dimensions of optical fibers as highly sensitive sensors within the generic microchannels [30,31,32]. The combination of microfluidics and fiber-based sensors allows for the separation and enrichment of analytes before optical analysis, helping to eliminate matrix interference and improve the selectivity, sensitivity, and reproducibility of optical detection. Microchannels can provide a stable measurement environment and improve the stability of optical fiber sensors for optical detection. This integration prevents the oxidation or rearrangement of the sensing components that may occur when fiber-optic sensors are exposed to air for an extended period of time. This section focuses on fiber-optic sensors for optical detection on microchips, including Raman, UV-vis, fluorescence, and SPR/LSPR detection methods (Table 1).

#### 2.2.1. Raman Detection

Raman detection is less affected by water and air, making it widely attractive for the qualitative and/or quantitative analyses of analytes in complex environments. The intensity of Raman scattering is weak, typically solely 10^−12^ to 10^−6^ of the incident light intensity. The SERS technique can be employed, which amplifies a Raman signal greater than 10^6^ times by coating Au, Ag, and other noble metal nanostructures [49]. The SERS phenomenon can be explained as a surface effect. The Raman signal is greatly enhanced by a million times when the distance between the analyte and the rough surface of the metal as an SERS substrate is in the nanoscale. Currently, various strategies have been proposed for fabricating fiber-optic SERS sensors, including precious metal evaporation, the laser-induced deposition of nanoscale metals, and the assembly of metal colloidal nanoparticles [50]. For example, a fiber-optic SERS sensor was developed through hydrothermally growing AgNPs on the fiber-optic sensor with thiol functionalization, enabling the effective identification of the target 4-aminothiophenol with a low limit of detection (LOD) of 2.15 × 10^−11^ mol/L [50]. Strobbia et al. [51] designed a fiber-optic sensor whose surface was decorated with AuNPs@Ag nanoprobes by shaking, aimed at detecting miRNA based on the SERS-sensing mechanism of nanoprobes. Xia et al. [52] proposed an embedded SERS sensor in which AuNPs were fabricated on the inner surface of an offset-multimode fiber-capillary-multimode probe using the AuNP self-assembly method. Liu et al. [53] introduced a method called fiber-optic light-induced nanoparticle aggregation SERS to enhance the Raman scattering intensity at the end face of the collection fiber. In Liu’s method, the AuNP substrate in a solution was driven along the optical axis under the radiation pressure of a non-focus laser beam and accumulated at the end face of the collection fiber to form “hot spots”. This led to a 30-fold increase in the Raman scattering signals of the analyte Rhodamine B.

**Figure 3:**
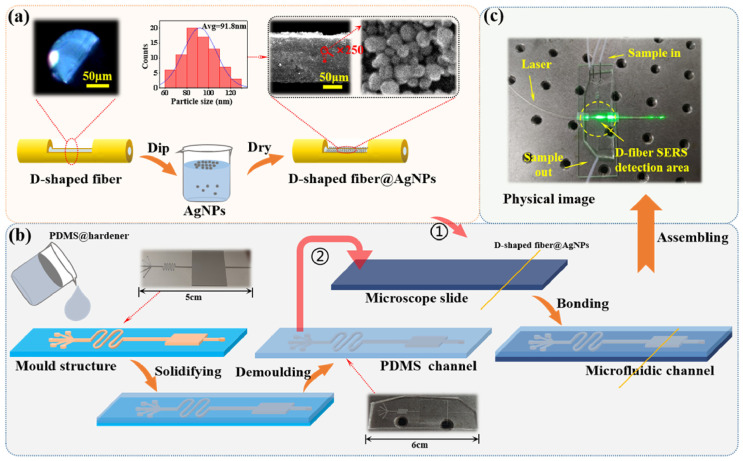
Experimental process flowchart. (**a**) Preparation process of the DSF-AgNP composite structure; (**b**) fabrication process of the microfluidics-integrated D-shaped optical-fiber SERS probe; (**c**) photograph of the fabricated sample. Reprinted with permission from [57]. Copyright 2022 Optica Publishing Group.

Sample preprocessing as well as mixing with nanoscale precious metals have become essential steps because of the potential contamination in practical situations outside of the laboratory. A fiber-based Raman system is integrated with lab-on-chips to ensure efficient nanostructures–sample blending or separating, enabling multiplex analysis and avoiding cross-contamination [54]. Choi et al. [55] achieved simultaneous bioaerosol sampling and SERS detection in real time by microfluidics. Airborne bacterial particles were carried radially outward in the curved channel under their inertia as passing through the μ-sampler. These bacterial particles were separated from air stream and further mixed with AgNPs in a serpentine channel that was used to accelerate the aggregation of bacterial particles and AgNPs. This work allowed AgNPs to directly bind to the cell wall of the bacterial particles in the microchannels. The designed device, with an LOD of 100 cfu/mL, achieved the integration of separation, enrichment, and measurement of samples in a microchip, which provided an important application prospect for the continuous environmental monitoring of suspended toxic components in the air. Zheng et al. [56] designed a near-infrared immunoassay biosensor for SERS detection using an optical-fiber-embedded microchip. The SERS probe was composed of a metal–organic framework and AuNPs, which were anchored with toluidine blue as an SERS tag that was linked to the analyte brain natriuretic peptide via the sandwich method. The AuNP substrate was glued to the bottom of the microchannel, which served as an enhancer as the SERS-tagged brain natriuretic peptide passed through the microchip. The amplified Raman signal was collected by the fiber-optic probe of a portable Raman detection system with an all-fiber-connected configuration. This sensor, with an LOD of 1 pg/mL, provided an effective scheme for the early diagnosis of chronic heart failure. Huang et al. [57] proposed a multi-microchannel microchip integrated with a D-type optical-fiber SERS probe structure that enabled the simultaneous detection of multiple molecules. To prepare a D-shaped optical-fiber SERS probe, a uniform AgNP layer was applied to the fiber using the liquid–liquid interface method, as shown in Figure 3a. A microchannel was produced through the use of a multichannel microfluidic template designed in-house (Figure 3b). The D-shaped optical fiber was then plasma-bonded to both the cover glass and multi-microchannel, thus resulting in an SERS probe (Figure 3c). The sample that was produced through this method demonstrated the excellent detection performance of Rhodamine 6G, boasting a low LOD of 10^−11^ mol/L and an enhancement factor of 1.14 × 10^9^.

Microfluidics coupled with fiber-optic sensors allows for the separation and enrichment of analytes in a microchip before Raman analysis, helping to eliminate matrix interference and improve the selectivity, sensitivity, and reproducibility of Raman detection. Fiber-optic sensors can be mounted directly on microchips to accurately transmit and collect the light signals, avoiding the need for sample relative calibration using confocal microscopes or complex supporting optical elements [58]. On the other hand, the prolonged exposure of fiber-optic sensors to the air is a latent factor of these internal nanoscale precious metals to be oxidized, which would reduce the Raman signal enhancement effect. Therefore, the on-site application of this sensing device will face significant challenges in terms of its operational lifetime.

#### 2.2.2. UV-Vis Detection

UV-vis detection is widely applied in the fields of agriculture, biomedicine, and environmental protection, including adulteration diagnosis, enzymatic assays, cell sorting, and dye monitoring [59,60,61,62]. UV-vis detection with a long optical path aims to enhance the sensitivity of detection. When it comes to the detection on microchips, the small area of microchannels as well as the short optical path limit the detection sensitivity. Researchers have combined microfluidics with fiber-optic sensors to extend the optical path by designing reflection absorption units, e.g., micro-cavities or resonator structures, which effectively improve the detection sensitivity using optical feedback [63]. For example, a miniaturized chip was incorporated with reflectors for the real-time detection of phosphate [63]. The probe light was fed into the microcavity through an optical fiber, where it was reflected multiple times between the reflectors to increase the optical feedback and was analyzed by the detector. The length of the absorption cell was reduced to 300 μm and the LOD reached 0.1 μmol/L. Meanwhile, the detection time was shortened from 20 min to 6 s. In addition, Choi et al. [64] designed an in-plane detection system to collect the greater width of the droplets on the digital microfluidic device, which defined the optical absorption path. The source/collection fiber was aligned in the plane of digital microfluidics by a custom manifold, achieving the non-contact optical measurement through the width of a droplet. This custom manifold allowed the insertion and alignment of fiber-optic sensors in seconds, permitting the rapid adjustment of the path length among the fiber-optic sensors. The improvement in the detection sensitivity was achieved by the increased optical path and “liquid lensing” effect, in which the presence of a droplet with a larger width between the fiber-optic sensors increased the fiber-to-fiber transmission of light by ∼2× through refraction and internal reflection.

**Figure 4:**
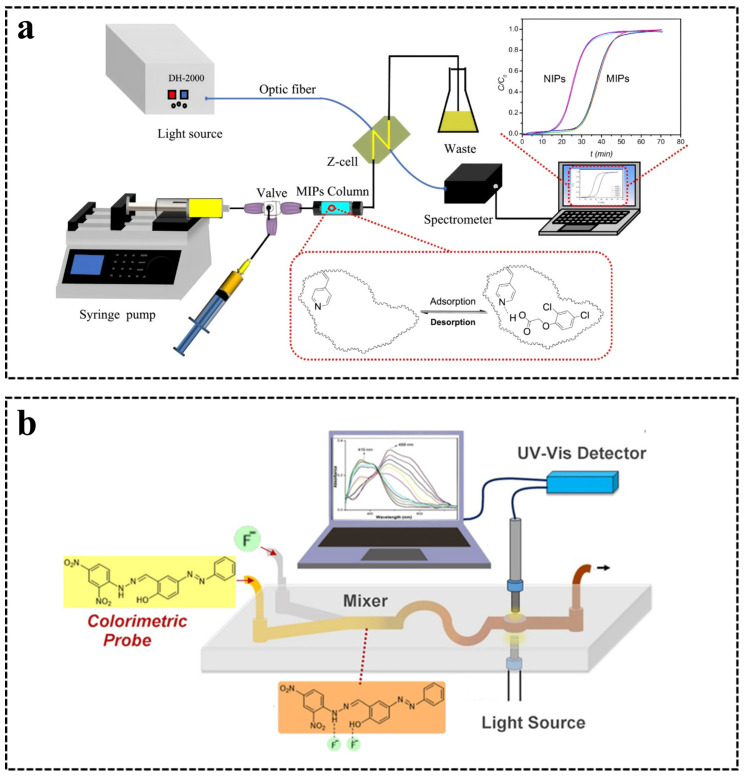
(**a**) Schematic illustration of the online measurement system of fixed bed adsorption. Reprinted with permission from [66]. Copyright 2020 Elsevier B.V. (**b**) Schematic of an optofluidic lab-on-a-chip device for the real-time analysis of fluoride ions combining the newly developed colorimetric probe, a PDMS microfluidic channel with mixers, and fiber-optic UV detector. Reprinted with permission from [67]. Copyright 2021 Elsevier B.V.

A strategy generalized as “front-end separation and back-end detection” can also be used to achieve the UV-vis detection of fiber-optic sensors on microchips. That is, the analyst is separated, mixed, enriched, etc., before/on the inflow into the microchips, and then the optical path is extended by the designed shapes of the detection cell on the microchips, such as Z/U-shaped channel, to improve the detection sensitivity [65]. Hu et al. [66] proposed a strategy for the online monitoring of the fixed bed adsorption process by extending the optical path using a self-assembled fiber-optic sensing system. In Figure 4a, a Z-shaped cell was designed to connect waste with columns that were packed with molecularly imprinted polymer particles for the adsorption of 2,4-dichlorophenoxyacetic acid. An optical path of 10 mm length was constructed between two embedded fiber-optics on the flow cell for absorption detection. This method showed a fast adsorption rate and high adsorption capacity (28.50 mg/g) in measuring 2,4-dichlorophenoxyacetic acid in lake water samples. In 2021, Bhat et al. [67] achieved the sensitivity of fluoride ions based on microchips and fiber-optic sensors. The microchip was designed with an S-type channel to promote the mixing of the colorimetric probe and fluoride ions followed by a detection zone with a 10 mm path length (Figure 4b). The proximity fibers were aligned with the pre-designed detection zone perpendicular to the flow direction and were used to connect the light source with the detector. This microchip combined with a fiber-optic sensor showed an LOD of 0.79 mg/L.

Furthermore, great advances have been made in improving the detection sensitivity through the use of a liquid core waveguide and a micromachined fiber tip with a tapered microlens. Li et al. [68] created a fiber-optic-based microfluidic setup that was shown to be ultra-sensitive for monitoring aggregation-induced emission molecules. The microchannel relied on an ablated micro-hole and an untreated open end of the capillary. A fiber tip was embedded into a capillary and positioned near the end of the micro-hole. Tapered microlenses were utilized to enhance the coupling efficiency at the fiber-tip surface, while the capillary, with low refraction, reduced the transmission loss at the liquid–Teflon interface of the liquid core waveguide. This compact device had an LOD of 0.1 μmol/L, which had vast potential for biosensing and drug screening owing to its highly sensitive and small sample volume.

UV-vis detection in microchips coupled with fiber-optic sensors provides an alternative to conventional experimental systems for absorbance measurement. Some special microchip-based detection cells and fiber-optic sensors, such as Z/U-shaped channels and microfibers, solve the problems of short absorption cells and low detection accuracy in conventional detection processes. In addition, the strategy called “front-end separation and back-end detection” aims to improve the sensitivity of absorption detection by separating, purifying, enriching, etc. the analyte before analysis. This integrated device shows a stronger capability in size, cost, sample preprocessing, and detection sensitivity, which has proved to be suitable for on-site detection.

#### 2.2.3. Fluorescence Detection

Fluorescence detection on microchips is commonly performed by laser-induced fluorescence detection that focuses the excitation light on the testing region of the microchannels and acquires the excitation fluorescence signal via a photo-detector [69]. However, laser-induced fluorescence detection with a vast size is limited to laboratory use and is not conducive to device miniaturization. Compared with traditional fluorescence detection, fiber-optic sensors with a small size are easy to embed in microchannels, which achieves the combination of excitation and reception fluorescence without a complicated optical alignment and exhibits an excellent sensing effect on microchips [68]. For example, Li et al. [29] fabricated a tapered fiber-optic sensor with a waist diameter of 720 nm, which was inserted into a 125 μm microchannel and showed a high sensitivity to a Rhodamine 6G solution with an LOD of 100 pmol/L. Zhang et al. [70] designed a nanofiber with a waist diameter down to 300 nm, which could be integrated into a capillary microchannel with a diameter of 375 μm.

**Figure 5:**
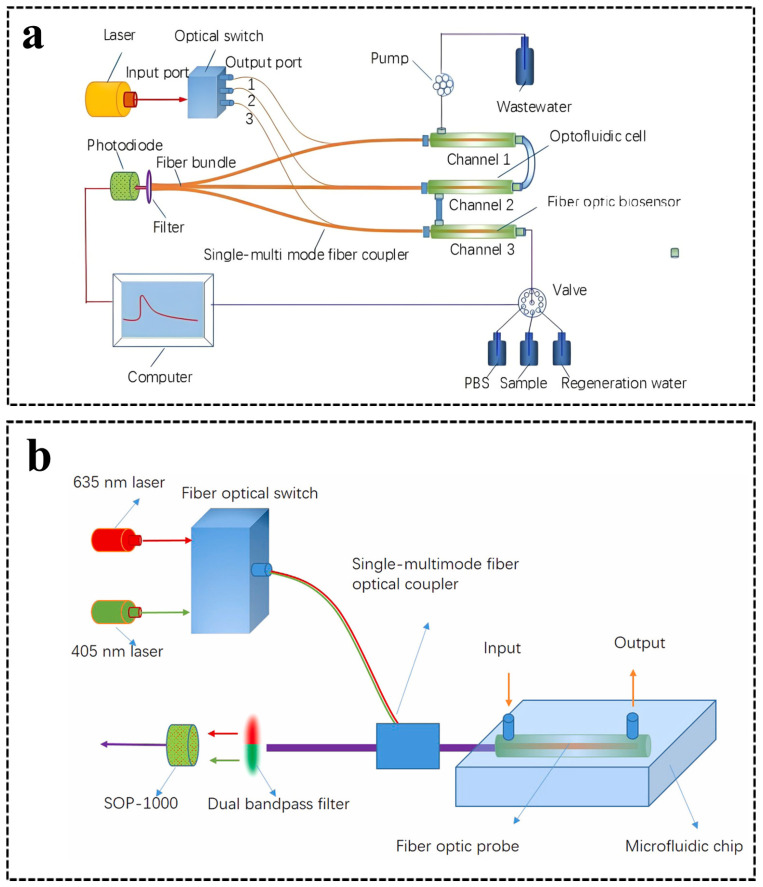
(**a**) Scheme of the multichannel all-fiber optofluidic biosensing platform system. Reprinted with permission from [72]. Copyright 2018 Elsevier B.V. (**b**) Scheme of the dual-color total internal reflection fluorescence-detecting platform. Reprinted with permission from [73]. Copyright 2019 Elsevier B.V.

Currently, high-throughput microchips integrated with fiber-optic sensors can be easily implemented for multi-beam and/or multi-wavelength fluorescence detection. Multiple fiber-optic sensors can be arranged in series or parallel sequences for collecting the fluorescence signals and guiding them to multiple detectors. In 2016, a compact biosensor based on microfluidic capillary action and a fiber-optic sensor was developed to achieve the aim of detecting multiple samples in one device [71]. Three capillaries with the same inner diameter of 536 μm were embedded into the microchip in parallel to form a three-to-one multichannel. The capillary was filled with a light blocker to form an annular chamber so that the light guided by fiber optics was only exposed along the rim of the capillary, aimed at preventing the light from directly illuminating the molecules inside the channel. This biosensor solely consumed 10 μL per sample and was used for measuring the analyte C-reactive protein with an LOD of 1.94 ng/mL. In 2018, Li et al. [72] designed a multichannel all-fiber optofluidic biosensing platform (M-AOB) that achieved the simultaneous measurement of up to three trace targets. Three microchips were sequenced in series for the simultaneous detection of multiple targets. The transmission of excitation light and fluorescence was performed by a 1 × 3 fiber-optic switch and three single-multimode fiber couplers on the M-AOB platform (Figure 5a). This M-AOB platform measured atrazine and 2,4-dichlorophenoxyacetic acid in water with LODs of 0.03 mg/L and 0.04 mg/L, respectively.

Unlike the M-AOB platform mentioned above, Song et al. [73] developed a dual-color total internal reflection fluorescence detection platform. The time-resolved effect of a fiber-optic switch was utilized to achieve the sensitive and simultaneous detection of fluorescent signals at two various wavelengths with a single photodetector (Figure 5b). The 1 × 2 fiber-optic switch controlled two-wavelength-excited lights to alternatively enter into the combined tapered fiber-optic probe, which achieved the alternating excitation of fluorophores cyanine 5.5 (405 nm) and Pacific blue (635 nm) close to the surface of the probe. The LODs of cyanine 5.5 and Pacific blue were obtained by this two-wavelength excitation platform as 0.05 nmol/L and 2.1 nmol/L, respectively. The effective sample volume was 40 nL, which was very suitable for the miniaturized total analysis system.

Fiber-based sensors achieve the combination of fluorescence excitation and reception on the microchips without complex optical alignment and show an excellent sensing effect on microchips. Meanwhile, high-throughput microchips have been developed to achieve the multi-beam and/or multi-wavelength fluorescence detection of samples on a chip, which greatly improves the detection efficiency that will be applied to the field of on-site detection in the future. In practice, the fluorescence detection of target substances is limited by their fluorescence signals or the presence of functional groups that generate fluorescence via derivative reaction. Furthermore, fluorescence detection is highly sensitive to interferences, e.g., quenching effects and background fluorescence. Thus, it is imperative to verify the existence of these disturbances and the extent to which they affect the sample determination in quantitative measurement.

#### 2.2.4. SPR/LSPR Detection

Fiber-optic surface plasmon resonance (FO-SPR) aims to reduce the SPR area to match the diameter of the fiber-optic sensor. In general, the sensing region ranges from a few mm to 1 cm in length on a fiber-optic sensor with a diameter of 400 µm or smaller [74]. Fiber-optic-localized surface plasmon resonance (FO-LSPR) is obtained by modifying metal nanostructures on a fiber-optic sensor. Embedding FO-SPR/LSPR sensors in microchannels aims to reduce the non-specific adsorption of sensors by optimizing the integrated structure of fiber-optic sensors and microchannels to control the flow rate of microfluidics [69]. Microchips provide a stable measurement environment for FO-SPR/LSPR, avoiding the rearrangement of nanostructures caused by the surface tension on the surface of sensors when fiber-optic sensors are exposed to air for a long time. Fiber-optic plays the role of a sensing element in on-chip detection, making it possible to sense the measure of samples into the microchannels by designing various structures, such as a tapered fiber, curved D-type fiber, and micro-structured hollow fiber. A type of microchip coupled with a D-structure fiber-optic sensor was proposed [75]. The sensing part of the D-type sensors was polished and then coated with a gold film with a thickness of 10–12 nm by sputtering to excite the evanescent field and SPR. The refractive indices of liquids, e.g., ethanol, methanol, and glucose solutions, were measured using the D-type sensors with a sensitivity of 10^−5^ RIU. Yang et al. [76] proposed the hollow silica capillary structure of the FO-SPR sensor in a microchip for measuring the refractive index. A segment of the hollow silica capillary was spliced between two multimode fibers, which constituted a compact hetero-core sensing structure. This FO-SPR sensor coated with a 60 nm thickness of gold was demonstrated to have a sensitivity of up to 7225.63 nm/RIU.

**Figure 6:**
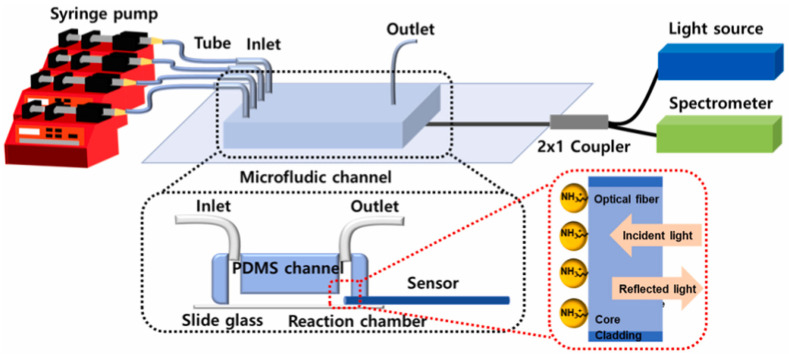
A sandwich immunoassay using second antibody–second AuNP conjugates was combined with FO-LSPR for the real-time analysis of low-concentration thyroglobulin in serum. Reprinted with permission from [79]. Copyright 2022 Elsevier B.V.

Several factors have an impact on the SPR/LSPR effectiveness, including the composition, size, geometry, and dielectric environment of metal films or nanoparticles [77]. Metal nanoparticles are most commonly used as sensing substrates, and their particle size affects the excitation to generate LSPR. Kim et al. [78] combined an FO-LSPR sensor with a microchannel, which was applied to measure the different concentrations of a prostate-specific antigen (PSA). This FO-LSPR sensor was fabricated by immobilizing AuNPs with a 50 nm diameter on the surface of the fiber-optic sensor. An anti-PSA was immobilized on the AuNP surface by electrostatic attachment and used to capture the PSA in an antigen solution. In this scheme, the PSA was measured with an LOD of 124 fg/mL. Similarly, a sandwich immunoassay using second antibody–second AuNP conjugates was combined with FO-LSPR for the real-time analysis of low-concentration thyroglobulin in serum (Figure 6) [79]. The sensing part of the cross-section of the fiber-optic sensor was hydroxylated, and immobilized AuNPs with a 55 nm diameter by electrostatic attachment. This FO-LSPR sensor was embedded in a microchip to avoid contamination from the external environment and the evaporation of samples on the sensor surface. When a sandwich strategy was formed using a second antibody–second AuNP signal amplifier, the LOD was enhanced 15 times (6.6 fg/mL). In 2022, Jyoti et al. [80] investigated adulterated urea in milk using metal oxides as fiber-optic sensing substrates into microchips. A ZnO nanorod structure, whose length was in the range of2.4–2.6 µm and diameter ranged from 190 nm to 205 nm, was deposited on the uncladded region of the optical fiber sensor via a hydrothermal process to generate LSPR sensing. An additional urease layer was coated on ZnO-based fiber-optic sensor, and then this sensor was placed in a flow cell to check the enzymatic reaction with urea that was adulterated in milk. Quantitatively, this sensor had a sensitivity of 0.084 nm/mmol, which was about 8-fold greater than the non-enzymatic sensor. Moreover, it had a rapid response speed of 5–7 s, which enabled it to address health problems like kidney failure and liver damage. In addition, a polymerase chain reaction chip integrated with an FO-SPR sensor coated with an asymmetric bimetallic (Ag/Al) layer was regarded as a DNA amplification-to-detection instrument for the generation and detection of the DNA amplicon of *Salmonella* spp. [81]. The detection chamber inside the chip of 1 cm (length) × 2 mm (width) × 1 mm (depth) was equipped with an SPR fiber sensor head, and a serpentine microchannel with a total length of 2.3 m was used for DNA molecule amplification. The amplification reaction of DNA of *Salmonella* spp. was performed on a chip with 30 thermal cycles in 30 min. To further improve the portability of the device, a power-free microchip coupled with a fiber-optic particle plasmon resonance biosensor was proposed to detect the SARS-CoV-2 N-protein [82]. The fluid was loaded into a perpendicular straight channel and passed through the detection chamber under gravity. The fiber-optic sensing region was decorated with a single-stranded DNA aptamer via amine coupling with carbonylated AuNPs, which was inserted into the detection chamber parallel to the flow direction. This power-free device exhibited a short assay time (15 min) and an LOD of 2.8 nmol/L, which allowed self-pumping by gravity without being connected to an external power source and enabled the loading and detection of multiple liquid samples.

**Figure 7:**
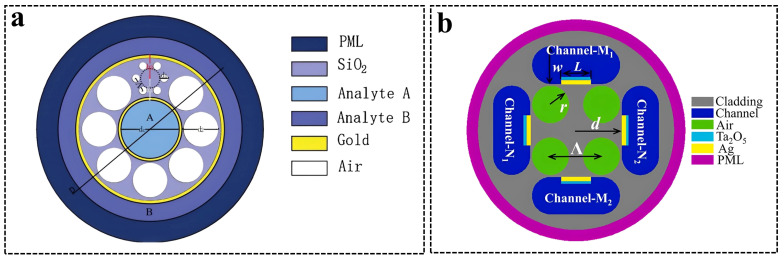
(**a**) Scheme of a PCF-SPR sensor with hexagonal air holes. Reprinted with permission from [84]. Copyright 2020 Elsevier B.V. (**b**) Scheme of a PCF-SPR sensor with square air holes. Reprinted with permission from [85]. Copyright 2022 Elsevier B.V.

Photonic crystal fibers (PCFs) have become a promising candidate for SPR sensing. taking advantage of their small size, ease of optical emission, single-mode propagation, and ability to control evanescent field penetration [83]. PCFs consist of a core and cladding similar to the traditional fiber-optic sensor, but the cladding region has periodic air holes that manage light propagation. PCFs have the advantage of flexibility in design, whose geometry can be optimized for achieving the optimum evanescent field, such as hexagonal, square, and octagonal. Yao et al. [84] proposed a dual-sample simultaneous detection sensor using an up-fiber core PCF (Figure 7a). The upward-shifted structure of the fiber core enlarged the air holes, and the liquid channels to be tested in the large air holes were favorable for coating a gold film and filling liquid. This structure was without the characteristics of rotational symmetry, which greatly reduced the fiber confinement loss and had a high sensitivity of 8300 nm/RIU. In addition, a novel PCF-SPR sensor with two orthogonally polarized core modes was designed to achieve the simultaneous measurement of the magnetic field and temperature [85]. Four annular microchannels coated with Ag-Ta_2_O_5_ films were symmetrically distributed in PCF cladding. In Figure 7b, the upper/lower microchannels had magnetic fluids, and then the temperature-sensitive material penetrated the left/right microchannels. The refractive index of magnetic fluids was strongly dependent on the magnetic field and temperature, while the refractive index of temperature-sensitive materials was only affected by the temperature. Thus, the information on the magnetic field and temperature carried by two orthogonally polarized core modes could be separated. The designed sensors showed a magnetic field sensitivity of 265 pm/Oe and a temperature sensitivity of 1410.7 pm/°C.

FO-SPR/LSPR sensors combine the high sensitivity of SPR/LSPR effect with the properties of fiber-optic sensors, such as being small in size and easy to functionalize, which promotes the development of integrated microfluidic sensor chips. Different structures of fiber-optic sensors have been designed to make it possible to detect the target based on the SPR/LSPR in the microchannels, which increases the SPR/LSPR detection range. The advantages of the FO-SPR/LSPR sensor system include the continuous monitoring of dynamic chemical reactions and the ability to detect turbid, opaque, or colored solutions. Nevertheless, non-specific adsorption remains a common issue with SPR/LSPR-based sensors. To address this problem, the integrated structure of fiber-optic sensors and microchannels may be further optimized to control the flow rate of microfluidics.

## 3. Applications

On-chip detection technology enabled by microfluidics coupled with fiber-optic sensors plays an essential role in many fields. Microfluidics offers a miniaturized platform for the fast analysis and detection of a small volume of fluid samples. The integration with fiber-optic sensors combining the advantages of optical detection or sensing measurement further develops the application of microfluidics. The miniaturized detection device is attractive in field detection for biological analysis, food security, and environmental monitoring. In this review, the current status of these integrated device strategies is introduced in various scenarios.

### 3.1. Biological Analysis

Microfluidics combined with fiber-optic sensors overcomes the disadvantages of long analysis time, multistep processing, and the high cost of traditional analytical means, e.g., enzyme-linked immunosorbent assay, radioimmunoassay, and chemiluminescence analysis (CLIA), and is becoming a useful tool for detecting different biochemical substances, such as nucleic acids, proteins, and bacteria.

Nucleic acid analysis is becoming a key technique for disease diagnosis, genetic disorders, and pathogenic infections, which plays a crucial part in genetic engineering, drug screening, and clinical medical research [86]. Ngo et al. [87] proposed a biosensing method to recognize gene mutations by integrating the MutS protein from bacteria with a fiber-optic particle plasmon resonance sensing system. MutS proteins bound to AuNPs and deposited on the core surface of the fiber-optic sensor to generate nanoplasmonic absorption. A microchannel with 800 μm (width) × 800 μm (depth) was used for housing a fiber-optic sensor. This micro-device allowed for a short analysis time of 15 min, while the LOD of the analyte double-stranded DNA containing an A- and C-mismatched base pair could be as low as 0.49 nmol/L. Protein assay plays a crucial role in proteomics studies, which have important research implications for clinical diagnosis and disease status [88]. Kim et al. [89] proposed a simple FO-LSPR sensor for the rapid measurement of thyroglobulin. The sensing region located on the fiber end-face was composed of AuNPs with a 55 nm diameter and attached to the microchannel, whose height and width were designed as 250 µm to accommodate it. The straight channel formed between the reaction chamber and the inlet ensured that the continuous fluid was not exposed to the external environment. This proposed method with an LOD of 93.11 fg/mL achieved thyroglobulin detection in serum samples within 10 min. Compared to CLIA, the LOD of this approach was approximately lower by 100 times and the consumption time was shorter by approximately 6–120 times.

**Figure 8:**
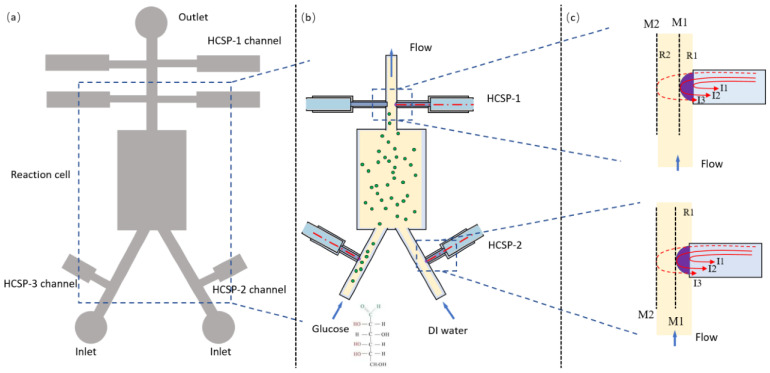
(**a**) Schematic diagram of the Y-shaped channel designed on the chip. (**b**) Optical fibers inserted into the fiber channels at the cross-section. (**c**) Principle of light field interference of the hemispherical cap sensor probe. Reprinted with permission from [91]. Copyright 2023 PDMI.

In addition, Hussain et al. [90] proposed an integrated approach of immunomagnetic separation, optical scattering, and machine learning for the rapid detection of *P. aeruginosa*. Three fiber-optic sensors were embedded in a microchip to connect a laser and two photodetectors. The laser light was aligned with the channel so that the light passed through the sample, and the scattered light was captured by photodetectors that were arranged symmetrically at 45° to the channel via fiber-optic sensors. The classification model built using a support vector machine algorithm achieved 97.9% classification accuracy. This method quantitatively detected *P. aeruginosa* with an LOD of 10^2^ CFU/mL and classified *P. aeruginosa* within 10 min. Qu et al. [91] introduced dual-parameter optical sensors with fiber tips into the microchips, enabling the continuous monitoring of the concentration and temperature of the fluids inside the microchannels. The Y-shaped channels with 600 μm width were used as the inflow channels for glucose and water, and it was connected to the central reaction cell to ensure homogeneous blending (Figure 8a). The channels on both sides of the Y-shaped could distribute the multiple fiber-optic sensors to monitor the concentration and temperature of the microfluid in real time. The temperature and concentration of the liquid could be monitored by simply embedding the fiber tip in the channel (Figure 8b). In this scheme, a temperature sensitivity of up to 314 pm/°C and a glucose concentration of up to -0.678 dB/(g/L) were achieved. This integrated setup is beneficial for drug discovery and pathological research and has an application potential for a miniaturized total analysis system.

### 3.2. Food Safety

The safety and quality of food are challenged due to its diversity. Therefore, there is an urgent need for the rapid analysis of food, preferably using on-site detection. The common detection objects in food include pesticides, fungicides, and microorganisms. Xiong et al. [92] created an optical sensor for measuring fluoride in tea to estimate its risk of fluorosis for public health. The optical sensor was assembled by inserting a decladding silica fiber-optic sensor into a transparent capillary tube. The ring microchannel was formed between the fiber sensor and capillary, which serves as both a sample flow cell and a detection cell. A narrow microchannel, with an inner diameter of 50 µm and a small volume of 1.2 μL, facilitated the solution displacement and shortened the response time (0.41 min). Compared to cumbersome and expensive spectrophotometry and chromatography, this sensor, with an LOD of 3.5 μg/L, offers a flexible tool for the in situ and real-time measurement of fluoride. A novel microfluidic system coupled with buried fiber-optic sensors was used to detect the viral pathogens of Phalaenopsis spp. [93]. A horizontal layout of buried fiber-optic sensors was applied to enhance the light signal, and an integrated micro-stirring device was used to uniformly distribute the magnesium pyrophosphate precipitate generated by the reverse-transcription loop-mediated isothermal amplification reaction. The changes in the optical signal caused by turbidity could be collected via optical fiber, enabling the direct on-chip detection of reverse-transcription loop-mediated isothermal amplification reaction products.

Directly or indirectly doping or the blending of external substances in food can change the biochemical properties and taste of food, as well as negatively affect the nutritional values. Vikas et al. [94] designed an FO-SPR sensor to identify the adulteration of glucose and fructose in pure honey. The 50 nm Ag film and graphene oxide were coated on the unclad fiber-optic portion to form the SPR-sensing region. The FO-SPR sensor was mounted on the flow cell, perpendicular to the direction of flow. This method showed that the fiber probe coated with the Ag film and graphene oxide had an enhanced sensitivity to identify the adulteration of glucose and fructose in the honey samples, at 24% and 37%, respectively. Yazdi et al. [95] successfully achieved the multiplexed detection of three highly regulated aquaculture fungicides. A microchannel was packed with silica microspheres that formed a porous matrix to concentrate AgNPs and adsorbed analyte molecules. Fiber-optic sensors were inserted in the microchannels and aligned to the detection region. The sample droplet was loaded at the inlet in a few seconds by using a pipette and applying negative pressure from the outlet. This device was capable of simultaneously measuring as low as 5 mg/L for methyl parathion, 0.1 μg/L for malachite green, and 5 μg/L for thiram. Oscar et al. [96] developed a simple micro-device that was used to determine the content of polyphenols in white wines. In this scheme, the reagents and samples were mixed uniformly via a serpentine channel with a total length of 4.61 cm. Optical fibers were perpendicularly inserted into the flow direction. Two optical fibers were inserted into a Z-type flow cell and were perpendicular to the flow direction. The two optical fibers were arranged facing each other with a tip distance of 7 mm as the optical path length, which increased the sensitivity during the absorbance detection. This method, incorporating a microchip, allowed the determination of polyphenols in white wines in 20 s, with an LOD of 0.016 mmol/L. Therefore, this method could be used for on-field measurements on account of its portability, low sample amount needed (3.4 μL), and fast analysis time (20 s).

### 3.3. Environmental Monitoring

As the exposure to contaminants in the environment increases, the monitoring requirements to understand and manage their risks to human health and the environment increase correspondingly. Thus, there is an urgent need of on-site detection methods with accuracy, rapidity, and convenience for the early detection of pollution incidents and to take remedial action when required. Microfluidics combined with optic-fiber sensors shows great advantages in size, flexibility, portability, response speed, operability, etc., and is suitable for the on-site analysis of the contaminant in liquid systems, such as inorganic ions and organic chemicals.

Heavy metal ion pollutants are emitted into the environment from various anthropogenic activities, such as the industry and agriculture. Youngvises et al. [97] developed a microfluidics-based micro-flow injection analysis system for the measurement of Ag (I) in real water using C-phycocyanin extracted from cyanobacteria as a colorimetric reagent. The micro-flow injection analysis system consisted of a polymethyl methacrylate (PMMA) microchip that was sandwiched between two PDMS sheets for packaging and facilitated its integration with fiber-optic sensors for signal monitoring. This developed method, with an LOD of 25 µg/L, achieved a sampling frequency of up to 33 per hour. A multiple-channel chemiluminescence detection system based on a microfluidic paper-based analytical device (μPAD) and fiber-optic sensors was presented to achieve the subsequent measurement of Cr^3+^ in water [98]. Each μPAD was composed of six separate channels in a parallel alignment to improve the throughput of the analysis. The chemiluminescence luminol-H_2_O_2_ system was used for the determination of Cr^3+^ in multiple channels of μPAD that were placed in a plastic holder equipped with six fiber-optic sensors for chemiluminescence. This miniaturized detection device with an LOD of 0.02 mg/L had an analysis time of within 1 min per one μPAD to obtain six measurements of various concentrations with a precision of <6.5%.

**Figure 9:**
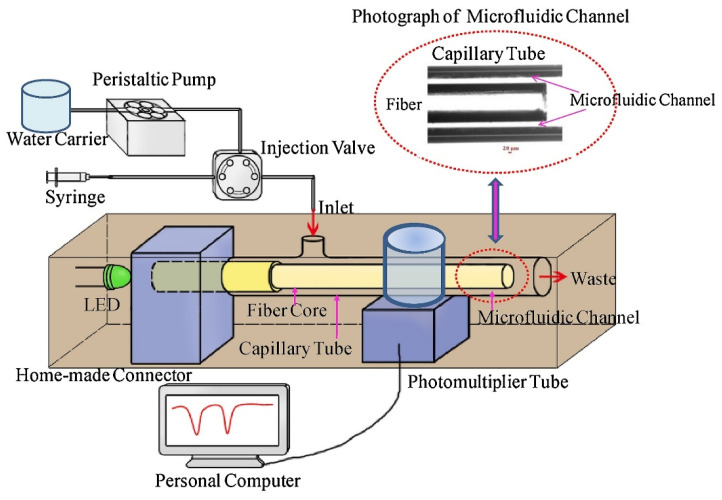
The whole evanescent wave set-up system. Reprinted with permission from [100]. Copyright 2020 Elsevier B.V.

The detection of anions in real water has also been reported. Prawpan et al. [99] developed a microfluidic-based method with front-face fluorometric detection, which was used for measuring inorganic iodine in natural water. The spiral microchannel was used to uniformly blend the As (III)/Ce(IV) reagents and the standard/sample solution. The online reaction of iodate into iodide for detecting the total inorganic iodine in water was performed on a microchip with a channel depth of 500 μm. This channel depth constituted the optical path for fluorescence detection. Front-face fluorometric detection from the fluorescing Ce (III) product was performed at the circular area of the microchip using a fiber that was positioned at the top of the chip and connected to a spectrofluorometer. The developed method, with an LOD of 7.7 μg/L, reached a level of throughput at 20 samples per hour, and is a potential tool for the rapid on-site detection of inorganic iodine in natural drinking water. In addition, a miniaturized sensor based on evanescent wave interaction has been developed for the colorimetric determination of harmful chlorine [100]. This sensor was made by embedding a decladding silica fiber into a transparent columnar tube. An annular microchannel was formed between the optical fiber and the capillary tube, which could be used both as a sample flow cell and a detection cell (Figure 9). The slit diameter (d_c_ = 50 μm) and micro-detection volume (1.2 μL) of the microfluidic channel greatly facilitated the colorimetric interaction of evanescent waves. The sensor was characterized by a small volume consumption (1.2 μL) and fast analysis speed (4.42 s). 

Ammonia nitrogen is a nutrient in water that can lead to eutrophication. Jiang et al. [101] presented a microchip without a complicated flow control system to achieve the on-site detection of ammonia nitrogen in water. To shorten the reaction time, a novel “flow and react regime” based on accelerated dissolution and reaction was proposed. The ammonia nitrogen-specific solid detection reagent was preloaded in the microchannel and compacted along the channel direction. As the fluids were introduced through the channel, capillary forces and localized turbulence accelerated the dissolution and reaction of the reagents, thus significantly enhancing mixing and producing shorter reaction times. Most of the colored solution was collected in the imaging cell; so, the resulting light signal could be quantitatively measured by fiber optics for changes in its chromaticity or absorbance. Ammonia nitrogen concentrations in the range of 0.3~10 mg/L were determined in 5 min using a chip spectrophotometer and a fiber spectrophotometer.

## 4. Conclusions and Future Prospects

More attention has been paid to developing an integrated system of microfluidic platforms and fiber-optic sensors for on-site detection in recent years. In this review, various optical detection methods available for chip detection via fiber-optic sensors are summarized, including Raman, UV-vis, fluorescence, and SPR detections. The applications of this integrated system in biological analysis, food security, and environmental monitoring are introduced in detail. The research direction of microfluidics integrated with fiber-optic sensors remains dedicated to automation, miniaturization, and multi-function to achieve automated analysis and improve the time efficiency of on-site detection. Currently, there are still relatively significant challenges that need to be addressed, which include the following: 

The sensitivity and stability of fiber-optic sensors on microchips face great challenges. When fiber-optic sensors composed solely of a single precious metal nanomaterial are exposed to the air for an extended period, it may cause the oxidation or rearrangement of the sensing components. Thus, there is a tendency to develop fiber-optic sensing materials with oxidation resistance to improve the lifetime of fiber-optic sensors for on-site detection. Microfluidic chips should be designed to optimize the structure of the chip so that it can be fully integrated with fiber-optic sensors to achieve a high-level integration of the detection system, increase the possibility of the simultaneous analysis of multi-samples on a single chip, as well as minimize non-specific adsorption. In addition, microchips integrated with fiber-optic sensors have limited advantages for single-parameter detection. High-throughput microchips integrated with fiber-optic sensors have been developed to achieve the multifunctional detection of samples on the same chip, such as multi-beam and/or multi-wavelength detection. Moreover, the design of fiber-optic sensing structures with different optical detection capabilities is integrated into a single fiber-optic sensor to achieve distributed measurement, which greatly improves the detection efficiency and ease for on-site detection. Considering that PCF-SPR can be configured to be reusable for uninterrupted monitoring, it has the potential to be developed into a promising tool for rapid lab-on-a-chip analysis for point-of-care diagnostics. To date, various structures of PCF-SPR sensors have been developed consisting of internal or external metal coatings. This complex structure limits the device implementation. Therefore, simple structures and uniform metal coatings should be designed for PCF-SPR sensors that can be applied for on-site detection.

## Figures and Tables

**Table 1:** Various optical detection methods for measuring the concentration of analytes using the integrated system of microfluidics and fiber-optic sensors.

Detection Method	Analyte	Sample	LOD (μg/L)	Time	Advantage	Ref.
Raman	Creatinine	Aqueous solution	1.13 × 10^2^	30 s	Short assay time/ Low sample consumption	[33]
	Rhodamine 6G	Water pollutants	4.79 × 10^−2^	——	High reusability/ Good controllability	[34]
	Levofloxacin	Tap water	3.61 × 10^3^	500 ms	Highly sensitive/ Reproducible	[35]
	Thiram	Aqueous solution	50	——	Portable/Small footprint	[36]
UV-vis	2-amino-4-chlorophenol	Chlorzoxazone	2.8	235 s	Fast pretreatment procedure	[37]
	Phenol	Aqueous solution	7.5	7 min	High sensitivity/ Rapid response time	[38]
	Tartrazine	Aqueous solution	2.67 × 10^3^	——	Low sample consumption/ Long absorption path length	[39]
	Chloramphenicol	Aqueous solution	5.0 × 10^−4^	30 s	Small footprint/ Low power consumption	[40]
Fluorescence	Hg^2+^	Real water	1.70	10 min	Portability/ Easy-to-operate	[41]
	Fluorescein dye	Droplets	3.32	——	High sensitivity/ Rapid response time	[42]
	Bisphenol A	Tap/Lake water	1.8 × 10^−2^	15 min	Miniaturization/ Flexibility/Mobility	[43]
	Dopamine	Human serum	4.59	60 s	Fast response/ High selectivity	[44]
SPR/LSPR	C-reactive protein	Serum	9.0	——	Easy to achieve/Real-time operation/ Label-free/Portable	[45]
	Protein	Urine	1.5 × 10^3^	——	Low consumption/ Highly sensitive detection	[46]
	Adalimumab	Plasma	350	12 min	Short assay time/ Low consumption	[47]
	Dengue virus NS1 protein	Blood serum	60	25 min	Easy lab-made set-up/Fast	[48]

## Data Availability

Data sharing is not applicable.
